# Rice genes involved in phytosiderophore biosynthesis are synchronously regulated during the early stages of iron deficiency in roots

**DOI:** 10.1186/1939-8433-6-16

**Published:** 2013-06-25

**Authors:** Reiko Nakanishi Itai, Yuko Ogo, Takanori Kobayashi, Hiromi Nakanishi, Naoko K Nishizawa

**Affiliations:** Graduate School of Agricultural and Life Sciences, The University of Tokyo, 1-1-1 Yayoi, Bunkyo-ku, Tokyo, 113-8657 Japan; Research Institute for Bioresources and Biotechnology, Ishikawa Prefectural University, 1-308 Suematsu, Nonoichi, Ishikawa 921–8836 Japan

**Keywords:** Early iron deficiency, Mugineic acid family phytosiderophores, Gene regulation network

## Abstract

**Background:**

The rice transcription factors IDEF1, IDEF2, and OsIRO2 have been identified as key regulators of the genes that control iron (Fe) uptake, including the biosynthesis of mugineic acid-family phytosiderophores (MAs). To clarify the onset of Fe deficiency, changes in gene expression were examined by microarray analysis using rice roots at 3, 6, 9, 12, 24, and 36 h after the onset of Fe-deficiency treatment.

**Results:**

More than 1000 genes were found to be upregulated over a time course of 36 h. Expression of MAs-biosynthetic genes, *OsIRO2*, and the Fe^3+^–MAs complex transporter *OsYSL15* was upregulated at the 24 h and 36 h time points. Moreover, these genes showed very similar patterns of expression changes, but their expression patterns were completely different from those of a metallothionein gene (*OsIDS1*) and the Fe^2+^-transporter genes *OsIRT1* and *OsIRT2. OsIDS1* expression was upregulated by the 6 h time point. The early induction of *OsIDS1* expression was distinct from the other Fe-deficiency-inducible genes investigated and suggested a functional relationship with heavy-metal homeostasis during the early stages of Fe deficiency.

**Conclusions:**

We showed that many genes related to MAs biosynthesis and transports were regulated by a distinct mechanism in roots. Furthermore, differences in expression changes and timing in response to Fe deficiency implied that different combinations of gene regulation mechanisms control the initial responses to Fe deficiency.

**Electronic supplementary material:**

The online version of this article (doi:10.1186/1939-8433-6-16) contains supplementary material, which is available to authorized users.

## Background

Iron (Fe) is essential for numerous biological oxidation–reduction reactions in plants. Despite its abundance in soils, Fe is present in the insoluble Fe(III) form under the oxidative soil environment and is not readily available to plants. The low solubility and availability of Fe in the soil solution often induces Fe deficiency in plants, especially in high-pH soils. Therefore, Fe deficiency is one of the biggest problems in crop production, reducing yields and quality. Higher plants have evolved two strategies to take up Fe from soils (Römheld and Marschner 1986). With the exception of graminaceous plants, higher plants reduce Fe^3+^ ions in soils using the ferric reductase FRO, and take up Fe^2+^ ions *via* the Fe^2+^ transporter IRT. Graminaceous plants absorb Fe^3+^ directly using the natural Fe^3+^ chelators named mugineic acid family phytosiderophores (MAs). MAs are biosynthesized in roots and secreted into the rhizosphere *via* a specific exporter of MAs (TOM1), and then the complex of Fe^3+^–MAs is taken up by an Fe^3+^–MAs transporter (YS1) on the surface of the root (Curie et al. 2001; Nozoye et al. 2011). *TOM1*, which encodes a major facilitator superfamily protein, was identified as a highly Fe deficiency-inducible gene in rice. Both TOM1 and its barley homolog, HvTOM1, cause the efflux of 2′-deoxymugineic acid (DMA), the primary molecule of the MAs (Nozoye et al. 2011). One of 18 members of YS1-like proteins in rice, OsYSL15, is involved in taking up Fe^3+^–DMA complexes from the rhizosphere, especially under Fe-deficient conditions (Inoue et al. 2009). The amount of MAs synthesized under Fe-sufficient conditions changes to meet plants’ growth requirements for Fe (Nomoto et al. 1987). Under Fe-deficient conditions, the amount of synthesized MAs increases dramatically. The synthesis of MAs is regulated at the transcriptional levels of the many types of genes that are related to the supply of the precursor of MAs and the actual synthesis of MAs (Higuchi et al. 1999,2001; Takahashi et al. 1999; Negishi et al. 2002; Inoue et al. 2003,2008; Kobayashi et al. 2005; Bashir et al. 2006). The methionine (Met) cycle, through which Met is supplied efficiently for ethylene production (Miyazaki and Yang 1987), also works for the production of MAs in graminaceous plants (Kobayashi et al. 2005; Suzuki et al. 2006). Promoter-GUS analyses revealed that in rice roots, the expression of MAs synthesis genes is increased by Fe deficiency in almost all tissues, whereas under Fe-sufficient conditions, their expression occurs mostly in vascular bundles (Inoue et al. 2003,2008; Bashir et al. 2006). Rice is also adapted to submerged paddy fields by taking up Fe^2+^ ions in the same manner as dicots and non-graminaceous monocots, in addition to the utilization of the Fe^3+^–MAs mechanism (Ishimaru et al. 2006). The expression of the rice Fe^2+^ transporter genes *OsIRT1*, *OsIRT2*, and *OsNRAMP1* is induced under Fe-deficient conditions (Ishimaru et al. 2006; Takahashi et al. 2011a, b). In addition to MAs and their precursor nicotianamine (NA), citrate and phenolics, which can act as metal chelators, have been confirmed to be important for Fe homeostasis in rice (Yokosho et al. 2009; Ishimaru et al. 2011).

The transcriptional responses to Fe deficiency, including MAs synthesis and Fe uptake, are regulated by some specific *cis* elements, such as the Fe deficiency-responsive *cis*-acting elements (IDE) 1 and 2 (Kobayashi et al. 2003,2005). IDEF1 and IDEF2, which specifically bind to IDE1 and IDE2, respectively, were identified as key transcription activators in response to Fe deficiency in rice (Kobayashi et al. 2003,2007; Ogo et al. 2008). IDEF1 is a B3 region-containing protein, and IDEF2 is a NAC-domain protein. Both *IDEF1* and *IDEF2* themselves show no response to Fe deficiency at the transcriptional level (Kobayashi et al. 2007; Ogo et al. 2008). An Fe deficiency-inducible gene encoding a basic helix-loop-helix (bHLH) transcription factor, OsIRO2, is under the control of IDEF1 and regulates the expression of genes such as the NA synthase genes *OsNAS1* and *OsNAS2*, *OsYSL15*, and *TOM1* (Ogo et al. 2006,2007,2011; Kobayashi et al. 2007). Overexpression of *OsIRO2* by the *cauliflower mosaic virus* 35S promoter results in increased OsIRO2 protein levels and high secretion of DMA from roots (Ogo et al. 2007). *OsIRO2*-overexpressing plants tolerated Fe deficiency in calcareous soils and grain yields were greater than for non-transformants, but no differences were observed between *OsIRO2*-overexpressing plants and non-transformants grown in normal soils (Ogo et al. 2011). Analysis of the *OsIRO2*-overexpressing plants indicated that transcriptional co-activators need to be activated by Fe deficiency to induce the expression of genes downstream of OsIRO2 (Ogo et al. 2007,2011). In contrast to OsIRO2, the regulation of MAs biosynthetic genes by IDEF1 is specifically restricted to early Fe deficiency than in the subsequent progressed state (Kobayashi et al. 2009). IDEF1 has characteristic histidine–asparagine repeat and proline-rich regions that are known for binding divalent metals including Fe^2+^. Through these metal-binding domains, the cellular metal ion balance caused by changes in Fe availability would be sensed by IDEF1 and switches the pattern of IDEF1 regulation from the early Fe-deficiency state to the progressed state (Kobayashi et al. 2009,2011).

The root is the organ where Fe uptake from the soils occurs, and it consumes Fe itself as well as sending Fe to the shoot. Most previous studies on Fe deficiency-induced genes have focused on the progressed Fe deficiency stage rather than on the onset of Fe deficiency. The primal responses to the onset of Fe deficiency at the transcriptional level are still unclear, not only in rice but also in other graminaceous plants. In this report, we carried out a 36-h time-course analysis of rice roots in the early stage of Fe deficiency using a 44 K microarray. We previously reported a time-course analysis of a 1-day span using a rice 22 K microarray, in which we discovered *OsIRO2* (Ogo et al. 2006). The expression changes observed in the previous work were more dynamic in shoots than in roots. For analysis of the expression changes in roots, a much shorter time span seemed to be suitable. Therefore, a time-course analysis was performed in a 3-h span within 3–12 h after the onset of Fe-deficiency treatment, and in a 12-h span within 12–36 h after the onset of Fe-deficiency treatment. We report the synchronous expression of the MA biosynthetic genes, *TOM1*, *OsYSL15*, and *OsIRO2*, and the unique expression patterns of *OsIDS1* and *OsIRT1*.

## Results

### Classification of upregulated genes in the time-course analysis

**Figure 1:**
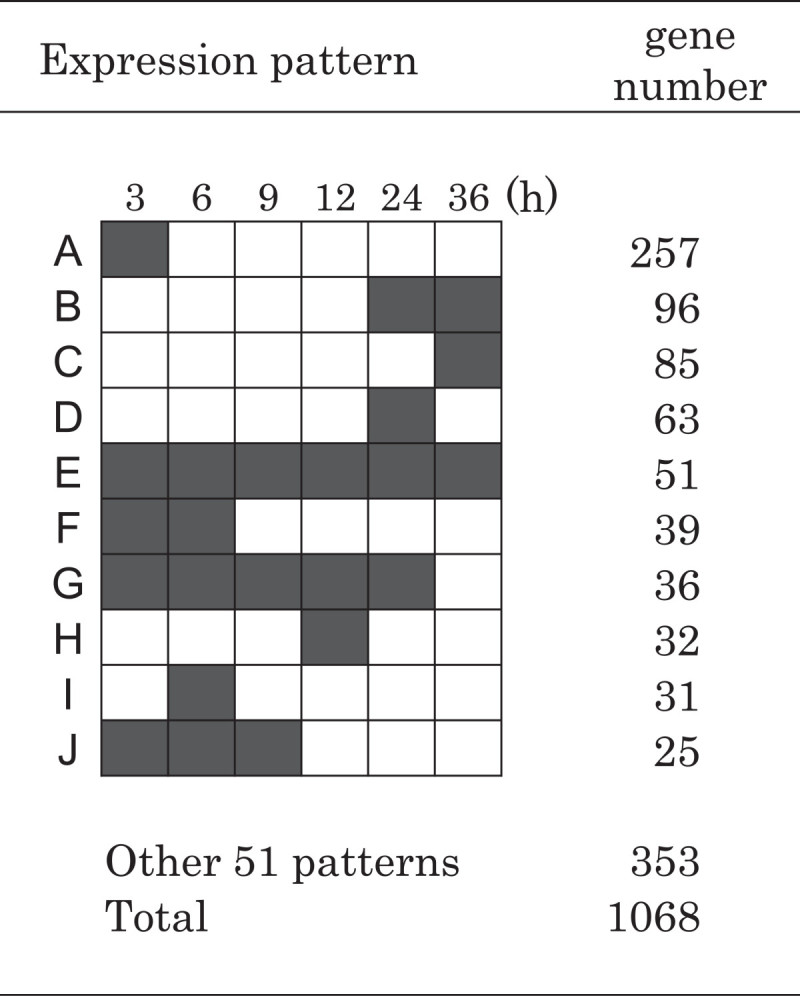
**Expression patterns of major groups of genes upregulated in the time-course analysis.** The 1068 genes upregulated by Fe deficiency were classified into 61 groups. The 10 largest groups were named groups A–J. Gray boxes indicate time points with upregulated gene expression and white boxes denote time points without upregulated gene expression.

Microarray analysis was performed using time-course samples taken at 3, 6, 9, 12, 24, and 36 h after the onset of Fe-deficiency treatment (Additional file 1). The expression of two transcription factors, IDEF1 and IDEF2, was stable at all time points, consistent with previous results from plants subjected to long-term Fe deficiency (Additional file 2;Kobayashi et al. 2007; Ogo et al. 2008). Genes whose expression ratio was over 1.95 at each time point were defined as upregulated. The numbers of upregulated genes at each time point were 551 at 3 h, 360 at 6 h, 295 at 9 h, 324 at 12 h, 434 at 24 h, and 388 at 36 h. The total number of upregulated genes during the 36-h treatment was 1068. The upregulated genes were classified into 61 groups according to their expression patterns, and the 10 groups with the largest probe numbers are shown with their expression patterns in Figure 1. The largest group (257 genes) from among all groups was upregulated only at 3 h (group A). In addition, more than 95 genes (groups F, I, J) were upregulated only during the first 9 h of Fe deficiency. Other groups with time-specific up regulation events that occurred only at 24 h or at 12 h (groups D and H) were also observed. In addition, some genes showed continued up regulation from the beginning of the Fe-deficiency treatment (groups E and G). Many genes participating in Fe uptake and Fe homeostasis were present in groups B and C, which were upregulated at 24 and 36 h or only at 36 h, respectively (Additional file 3). The Fe deficiency-inducible metallothionein (MT) gene *OsIDS1* was upregulated from 6 h.

### Fe availability decreases in the first 6 h of Fe-deficiency treatment

**Figure 2:**
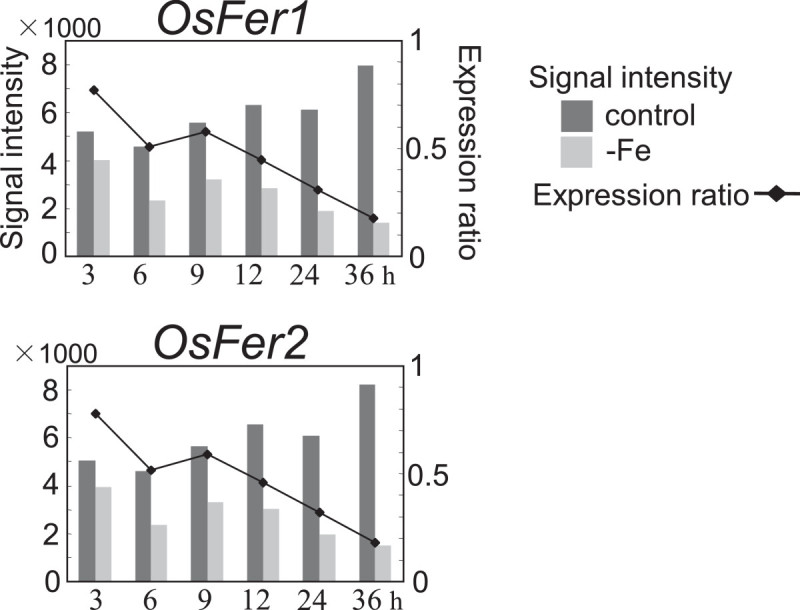
**Changes in expression of ferritin genes.**

Genes whose expression ratio was under 0.54 at any of six time points were searched out from the rest of the genes after selecting the upregulated genes. Three-hundred twenty-five genes on the array were defined as down regulated genes (data not shown). The most significant gene expression among them was that of ferritin genes, as the availability of Fe in cells would be deduced from the expression change of ferritin genes. The expression ratios of two ferritin genes, *OsFer1* and *OsFer2*, were below 1 at 3 h and then below 0.54 at 6 h (Figure 2), indicating that the decreasing Fe availability in root cells progressed quickly in the first 6 h of the Fe-deficiency treatment. Several genes encoding heme peroxidase family proteins were down regulated, although they have never been reported as related to Fe stress (data not shown).

### The expression of the MAs-related genes changed synchronously

**Figure 3:**
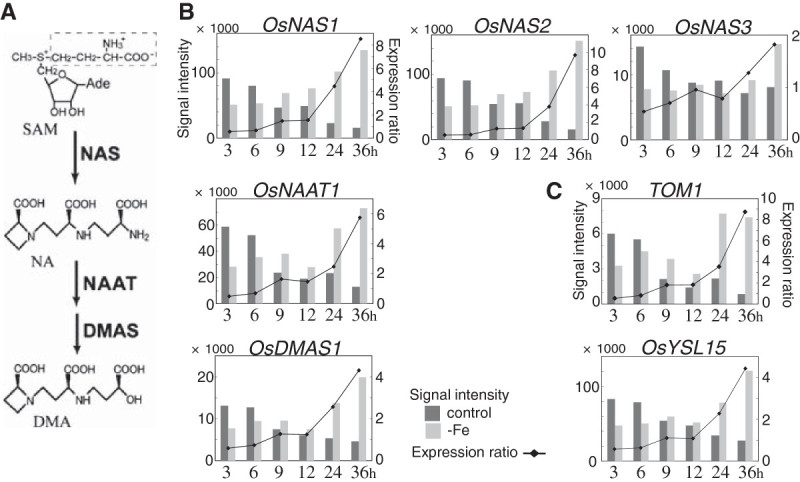
**Schematic pathway of the biosynthesis of MAs and expression changes in the related genes.** **A)** DMA is the sole MAs synthesized in rice. DMA is synthesized from *S*-adenosyl-l-methionine (SAM) by NAS, NAAT, and DMAS. **B)** Changes in the expression of MAs-biosynthetic genes. **C)** Changes in the expression of *TOM1* and *OsYSL15*.

The expression of genes encoding enzymes involved in MAs biosynthesis was well synchronized (Figure 3). The expression ratios of *OsNAS1*, *OsNAS2*, the NA aminotransferase gene *OsNAAT1*, and the DMA synthase gene *OsDMAS1* were below 1 at 3 h and 6 h, increased to around 1.5 at 9 h and 12 h, and finally surpassed 2 at 24 h and 36 h (Figure 3B). Unlike other genes involved in MAs biosynthesis, *OsNAS3* was not upregulated within 36 h of Fe-deficiency treatment, although increased *OsNAS3* expression was observed at 36 h (Figure 3B). As *OsNAS3* is expressed constitutively but suppressed by Fe deficiency in leaves, unlike the other two *NAS* genes (Inoue et al. 2003), its regulation in roots may be also different from that of the others. Transitions of their expression (except for *OsNAS3*) indicated by signal intensities were also similar, *i.e.*, the signal intensities of these genes increased according to the Fe-deficiency treatment in the Fe-deficient plants, whereas the rather high signal intensities at 3 h gradually decreased during 36 h in the control plants (Figure 3B). The expression patterns of the MAs exporter gene *TOM1* and a MAs-metal transporter gene *OsYSL15* were also similar to those of MAs-biosynthetic genes (Figure 3C).

**Figure 4:**
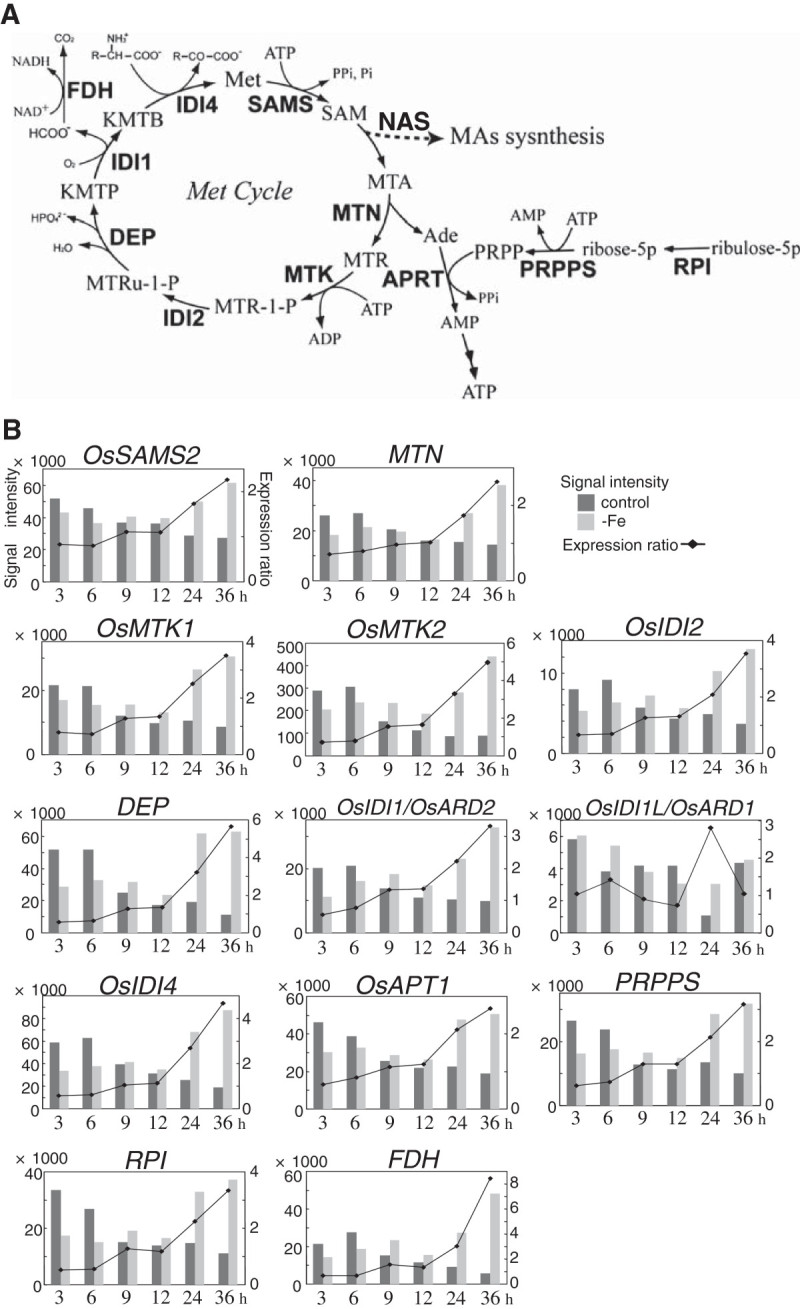
**Putative scheme of SAM supply for MAs synthesis and expression changes in the involved genes.** **A)** Biosynthetic pathway of the Met cycle and its collaborative pathways. Enzymes are shown in bold. MTA, methylthioadenosine; MTR, methylthioribose; MTR-1-P, methylthioribose-1-phosphate; MTRu-1-P, methylthioribulose-1-phosphate; KMTP, 1,2-dihydroxy-3-keto-5-methylthiopentene; KMTB, 2-keto-4-methylthiobutyrate; PRPP, phosphoribosyl pyrophosphate; SAMS, SAM synthetase; MTN, methylthioadenosine nucleosidase; MTK, methylthioribose kinase; IDI1, acireductone dioxygenase; IDI2, methylthioriburose-1-phosphate isomerase; DEP, dehydrase-enolase-phosphatase; IDI4, aspartate/tyrosine/aromatic aminotransferase; APRT, adenine phosphoribosyltransferase; PRPPS, PRPP synthetase; RPI, ribose 5-phosphate isomerase; FDH, formate dehydrogenase. **B)** Expression changes of Met cycle-related genes.

The Met cycle is known to supply *S*-adenosyl-Met efficiently, supporting MAs biosynthesis (Kobayashi et al. 2005; Suzuki et al. 2006). A scheme of the Met recycling and collaborative pathways is shown in Figure 4A. The expression of all genes that participate in the Met cycle and its collaborative pathways was upregulated from 24 h or 36 h after the onset of Fe deficiency (Figure 4B). Their transitions of signal intensities were very similar to those of MAs biosynthetic genes (Figures 3B and 4B): the signal intensities in the control plants decreased over the first 36 h while those in Fe-deficient plants increased. However, the *S*-adenosyl-Met synthetase gene *OsSAMS2* and the methylthioadenosine nucleosidase gene *MTN* were more slowly induced than other Met cycle-related genes and MAs biosynthetic genes. The expression ratios of *OsSAMS2* and *MTN* were below 1 at 3 h and 6 h, about 1 at 9 h and 12 h, and greater than 2 at 36 h (Figure 4B). Two genes encoding acireductone dioxygenase, *OsIDI1L/OsARD1* and *OsIDI1/OsARD2*, were both upregulated within 36 h of the onset of the Fe-deficiency treatment, but their expression patterns were completely different (Figure 4B). *OsIDI1L/OsARD1* was only upregulated at 24 h, whereas the expression pattern of *OsIDI1/OsARD2* was like that of other Met cycle-related genes.

### Search for genes expressed coordinately with MAs-related genes

We assumed that genes with the same regulation would show a similar expression pattern. Therefore, we searched for genes whose expression patterns were similar to that of the MAs biosynthetic genes, *TOM1*, *OsYSL15*, and the Met cycle-related genes (Figures 3BC and 4B) using the following three criteria: (i) they were upregulated (expression ratio ≥ 1.95) at 24 and 36 h of Fe deficiency or showed increased expression (expression ratio ≥ 1.55) at 24 h and were upregulated at 36 h, (ii) their expression ratios at 3 and 6 h were around 0.5 – 0.9, and (iii) their signal intensities in the control plants at 3 h were higher than those at 36 h, while their signal intensities in Fe-deficient plants at 3 h were lower than those at 36 h. Twenty-five genes satisfied all three criteria (Table 1). In particular, the bHLH transcription factor gene *OsIRO2* and the metal transporter gene *OsNRAMP1* showed similar expression to those of the MAs-related genes.

**Table 1:** **Genes with expression patterns similar to the MAs-biosynthetic and Met cycle-related genes**

	Gene locus	Definitions		Expression ratio					
				3 h	6 h	9 h	12 h	24 h	36 h
**Metabolism**									
	Os06g0639800	Cytochrome P450 family protein		0.70	0.69	1.16	0.87	7.91	*3.27*
	Os08g0562100	Sorghum chloroplast malate dehydrogenase-like (Fragment)		0.80	0.86	1.05	1.03	1.66	2.53
	Os09g0536700	Nodulin-like domain containing protein		0.77	0.83	1.29	1.31	2.45	3.51
	Os10g0440000	Cytochrome P450 family protein		0.73	0.83	0.85	0.80	2.48	3.17
**Membrane protein**									
	Os01g0878700	Amino acid transporter family protein		0.71	0.76	1.41	1.49	3.48	4.19
	Os03g0828600	Sodium/hydrogen exchanger family protein		0.48	0.75	1.23	1.47	*2.78*	5.78
	Os07g0258400	Metal transporter OsNRAMP1		0.66	0.89	1.30	1.24	2.93	4.34
	Os09g0440700	Copper transporter OsCOPT7		0.76	0.83	1.00	1.00	1.55	1.96
	Os12g0132500	MFS family protein TOM1-like		0.59	0.79	1.72	1.68	2.95	7.50
**Transfactor**									
	Os01g0952800	bHLH protein OsIRO2		0.74	0.91	1.19	1.11	2.00	3.06
**Others**									
	Os01g0546100	DUF6 containing protein		0.42	0.65	1.18	1.53	5.81	3.48
	Os01g0647200	Non-protein coding transcript		0.71	0.83	0.92	0.79	3.10	6.02
	Os02g0445100	Auxin-responsive family protein-like		0.67	0.58	1.40	0.77	2.32	2.05
	Os03g0204900	Hypothetical protein		0.67	0.76	1.34	1.26	2.64	3.62
	Os03g0256200	Hypothetical protein		0.68	0.69	1.19	1.17	2.29	2.92
	Os04g0675000	DUF789 containing protein		0.81	0.78	1.77	1.81	2.80	4.28
	Os05g0519300	DUF506 containing protein		0.88	0.69	1.00	0.83	2.17	5.51
	Os05g0551000	HHE domain containing protein		0.75	0.83	1.00	0.99	1.88	2.17
	Os07g0253600	(No Hit)		0.67	0.69	1.20	1.17	2.28	2.94
	Os08g0291000	(No Hit)		0.66	0.69	1.20	1.18	2.35	2.93
	Os09g0130300	Conserved hypothetical protein		0.50	0.82	1.14	0.91	1.85	2.71
	Os09g0345300	Leucine-rich repeat protein		0.66	0.70	1.02	1.04	2.32	2.62
	Os11g0129600	ELM domain containing protein		0.69	0.85	1.04	1.15	1.81	2.29
	Os12g0126200	ELM domain containing protein		0.70	0.86	0.99	1.05	1.79	2.27
	Os12g0236100	Conserved hypothetical protein		0.75	0.88	1.16	0.99	1.82	3.68

Expression ratios in italics did not meet the selective criteria for signal intensities even when they were greater than 1.95.

### Expression changes in metal transporter genes upregulated in early Fe deficiency

**Figure 5:**
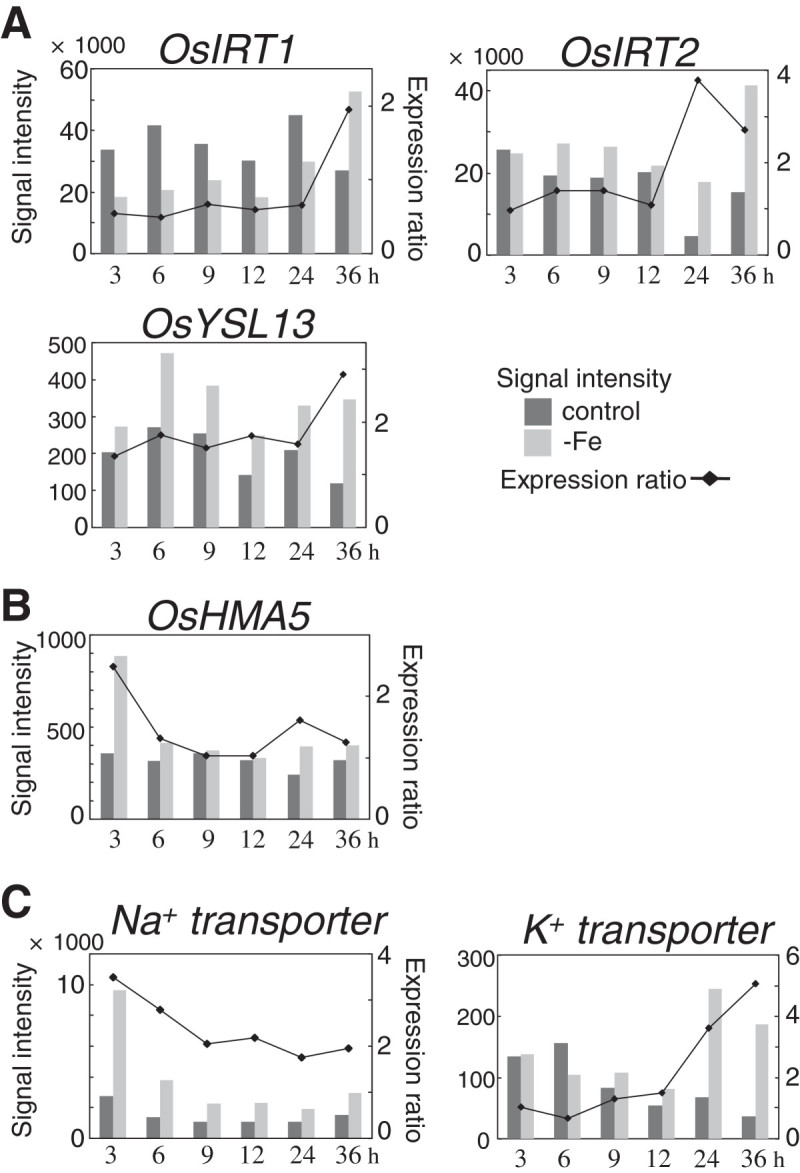
**Fe deficiency-inducible genes encoding functional or putative transporters.** **A)** Fe transporters. OsIRT1 and OsIRT2, Fe^2+^ transporters (Bughio et al. 2002; Ishimaru et al. 2006); OsYSL13, a putative MAs–metal complex transporter (Koike et al. 2004). **B)** Copper transporters. OsHMA5, a putative HMA-family protein (Lee et al. 2007). **C)** Monocation transporters. Na^+^ transporter, putative sodium transporter; K^+^ transporter, putative potassium transporter.

Several genes encoding functional or putative metal transporters were found to be upregulated. Among the genes encoding Fe transporters, five were upregulated. As previously described, *OsYSLl5* and *OsNRAMP1* were expressed in the pattern of the MAs-related genes (Figure 3C, Table 1). In contrast, the Fe^2+^ transporter genes *OsIRT1* and *OsIRT2* were expressed in patterns very different from those of the MAs-related genes and also from each other (Figure 5A). The expression ratio of *OsIRT2* was maintained at around 1 until 12 h of Fe deficiency and then was over 2 at 24 h and 36 h, whereas that of *OsIRT1* was maintained below 1 until 24 h and then reached 2 at 36 h. The putative MAs–metal complex transporter gene *OsYSL13* was upregulated only at 36 h (Figure 5A). The *OsYSL13* expression level was largely altered at each time point, although this did not influence its expression ratios. Among 18 genes of the rice YSL family, only *OsYSL15* and *OsYSL13* were upregulated at all of the six time points (data not shown). The copper (Cu) transporter genes *OsCOPT7* and *OsHMA5* were upregulated. *OsCOPT7* was expressed in a pattern similar to that of the MAs-related genes (Table 1), although it’s up regulation was observed only at 36 h. OsHMA5 belongs to the P_1B_-type heavy-metal ATPase (HMA) family that functions in transporting Cu (Lee et al. 2007). *OsHMA5* expression was upregulated only at 3 h (Figure 5B). Two monocation transporter genes were also upregulated. A putative Na^+^ transporter gene was highly upregulated at 3 h, after which its expression ratio gradually decreased to 2 (Figure 5C). In contrast, the expression of a putative K^+^ transporter gene was upregulated at 24 h and 36 h (Figure 5C).

### Expression of MT genes

**Figure 6:**
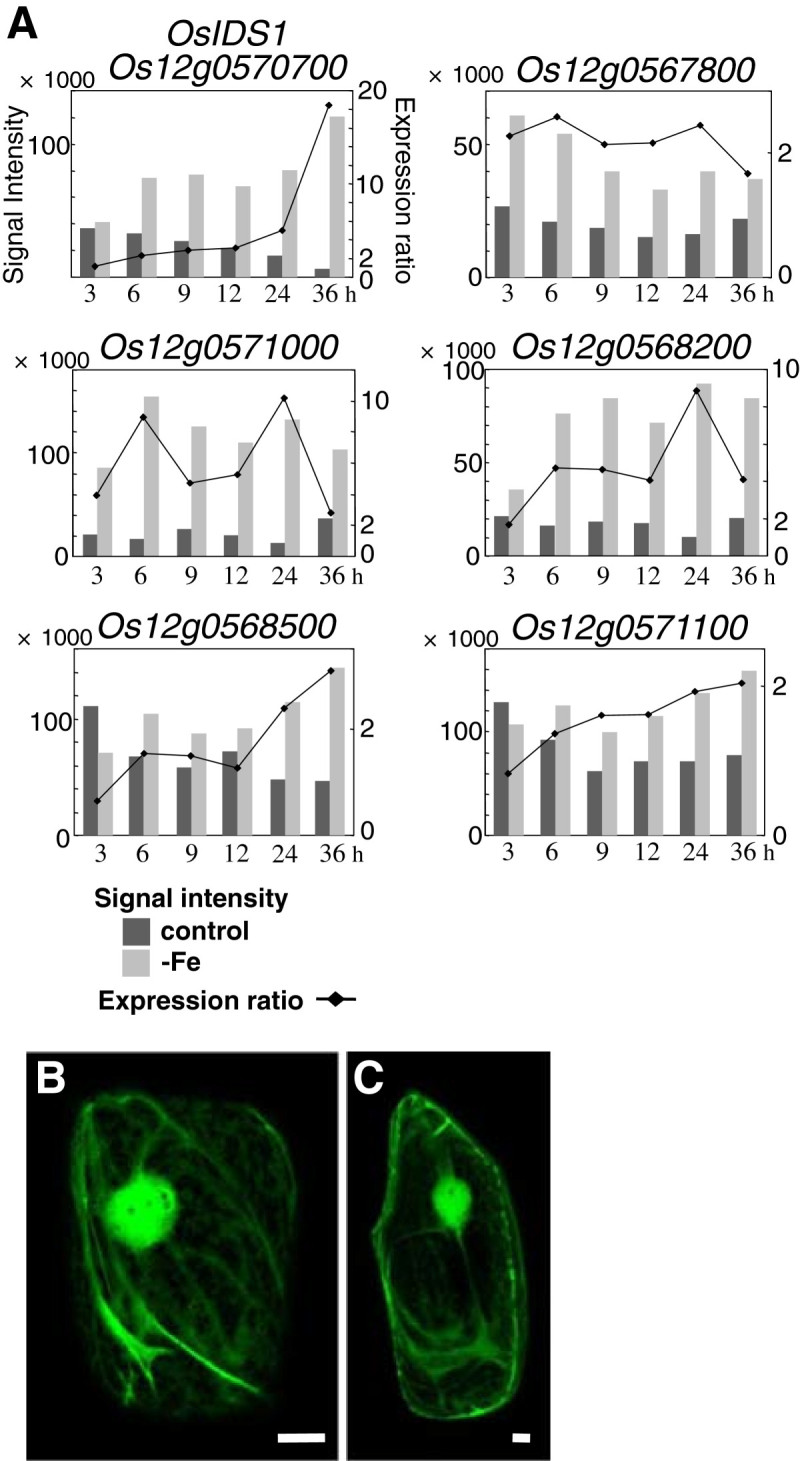
**Fe deficiency-induced MT genes.** **A)** Expression revealed by microarray analysis. **B**, **C)** Subcellular localization of OsIDS1. OsIDS1 proteins fused to the N terminus of sGFP were transiently expressed in onion epidermis cells. Bars indicate 20 μm. B, sGFP alone; C, OsIDS1-sGFP.

Six MT genes, including *OsIDS1*, were found to be upregulated by Fe deficiency (Figure 6A). MTs are small cysteine-rich proteins that generally bind zinc (Zn), Cu, cadmium, cobalt, and other metals. The changes in expression of the MT genes occurred much faster than the changes in expression of Fe transporter genes such as *OsYSL15* and *OsNRAMP1* and MAs biosynthetic genes (Figure 6). The six MTs belong to the class-I type of plant MTs (Zhou et al. 2006), and their genes are located in close proximity in the genome (data not shown). For the four MT genes *OsIDS1*, Os12g0567800, Os12g0571000, and Os12g0568200, up regulation began from the beginning of the Fe-deficiency treatment (3 h or 6 h). The expression ratio of *OsIDS1* began to increase from 6 h and kept increasing until 36 h (Figure 6A). The up regulation of Os12g0567800 and Os12g0571000 was maintained from 3 h to 24 h or 36 h, respectively. Os12g0568200 was upregulated from 6 h to 36 h. Os12g0568500 and Os12g0571100 were upregulated from 24 h and at 36 h, respectively.

The subcellular localization of MT proteins induced during the early stages of Fe deficiency can give an indication of their potential functions. A program that predicts protein subcellular localization (WoLF PSORT: http://wolfpsort.seq.cbrc.jp/) strongly predicted that barley IDS1 (Okumura et al. 1991), OsIDS1, and other MTs in Figure 6A localize to the chloroplast, where ferritin is known to localize. The subcellular localization of OsIDS1 was determined using synthetic green fluorescent protein (sGFP). An OsIDS1-sGFP fusion protein was mainly located in the cytosol in onion epidermis cells, just like sGFP alone (Figure 6B and 6C). OsIDS1-sGFP was not detected in subcellular compartments such as plastids. OsIDS1-sGFP was also not detected in chloroplasts (data not shown). We conclude that OsIDS1 localizes to the cytoplasm and not to plastids or chloroplasts.

## Discussion

### Met cycle-related genes were synchronously expressed with MAs biosynthetic genes

In total, 1068 genes were upregulated more than twofold at any time point in our time-course analysis (Figure 1). The observation of many time points revealed numerous differences in the responses of each gene to Fe deficiency. The expression patterns of more than 20 genes, which included MAs biosynthetic genes, Met cycle-related genes, *TOM1* and *OsYSL15*, were similar (Figures 3 and 4, Table 1). This synchronous expression of the Met cycle-related genes was especially interesting. The Met cycle supports not only MAs biosynthesis but also other SAM-consuming pathways, such as lignin biosynthesis and ethylene biosynthesis, even in rice roots (Miyazaki and Yang 1987; Ma et al. 1995). Their synchronous expression with MAs biosynthetic genes showed that the Met cycle is strongly regulated by Fe availability in rice roots, as we previously reported on the regulation of Met-cycle genes through *OsIRO2* (Ogo et al. 2007). This also was in accord with the case of the Met-cycle related genes formate dehydrogenase (*FDH*) and adenine phosphoribosyltransferase (*APRT*), which were hypothesized to be closely related to the Met cycle, even though they are not directly included in the Met cycle pathway (Figure 4;Suzuki et al. 1998; Itai et al. 2000). Consistently, *FDH* and the APRT gene *OsAPT1* showed synchronous expression with the Met-cycle and MAs-biosynthetic genes (Figure 4). In addition, genes encoding 5-phosphoribosyl pyrophospate (PRPP) synthetase were also found to be induced in barley roots by Fe deficiency (Nagasaka et al. 2009). Consistent with the high APRT activity in Fe-deficient roots of graminaceous crops (Itai et al. 2000), a PRPP synthetase gene was upregulated in the same expression pattern as other MAs-related genes in early Fe deficiency, strongly suggesting that the PRPP supply increased in response to increased adenine recycling. Feeding ^13^C-labeled ribose to Fe-deficient wheat resulted in ^13^C incorporation into MAs (Ma et al. 1995). The induction of PRPP synthetase genes by Fe deficiency in graminaceous plants explains why ribose is able to be easily introduced into the ribosyl group of ATP and converted into MAs. Furthermore, the ribose 5-phosphate isomerase gene *RPI*, which was one of the genes expressed synchronously with MAs-related genes and which we had hypothesized to be responsible for converting MTR-1-P into MTRu-1-P (Kobayashi et al. 2005), may support the PRPP supply by converting ribulose-5P into ribose-5P (Figure 4A).

### Genes synchronously expressed with MAs biosynthetic genes may have functions closely related to MAs biosynthesis and secretion

Precise mechanisms connecting the biosynthesis of MAs and the Met cycle are still unclear in many respects, including how to recycle AMP that is converted by APRT into ATP, how and from where to supply enough ATP to MAs biosynthesis under Fe-deficient conditions when mitochondria are also suffering from Fe deficiency (Mori et al. 1991), and how to transport SAM to the NAS proteins that are proposed to exist in MAs vesicles (Negishi et al. 2002; Nozoye et al. 2004; Nagasaka et al. 2005). There are likely to be more proteins that must be activated for vigorous MAs biosynthesis. These, as yet, unknown proteins may be encoded by some of the genes that are expressed similarly to MAs-related genes (Table 1) or those upregulated at 24 h and/or 36 h (groups B and C in Figure 1), whose functions in Fe deficiency are unclear.

Many potassium transporter genes were found to be expressed highly and constitutively in rice roots (data not shown). However, only one K^+^ transporter gene was induced in early Fe deficiency (Figure 5C). MAs are thought to be secreted by symport with potassium (Mori 1994; Sakaguchi et al. 1999). The Fe deficiency-induced potassium transporter gene could have a specific role in MAs secretion. Since root tips show vigorous MA secretion and Fe uptake (Mihashi and Mori 1989), the low signal intensities of this K^+^ transporter gene (Figure 5C) might reflect small numbers of the expressing cells, such as the epidermis and exodermis cells of root tips.

### Low Fe availability causes fluctuations in heavy metal balance and induces MT and metal transporter genes

Fe always competes with other metals for the activity of Fe transporters, such as IRT-, NRAMP-, and YSL-family proteins. Such competition is likely to be greatly influenced by Fe-deficiency treatment, especially under hydroponic culture conditions, since ions of Zn, manganese (Mn), and Cu exist abundantly in the culture solution, in contrast to the depleted Fe. Therefore, concentrations of Zn, Mn, and Cu in root cells are thought to increase immediately after the onset of Fe deficiency. In fact, the concentrations of Zn, Mn, and Cu increased in rice roots after a week of Fe-deficiency stress (data not shown). In the current work, decreased expression of two ferritin genes indicated that Fe availability in roots was already decreasing at 6 h (Figure 2). In addition, *OsHMA5*, which encodes a putative transporter that sequesters Cu into vacuoles (Lee et al. 2007), was transiently upregulated at 3 h (Figure 5B). This may reduce the Cu concentration in the cytosol. Some MT genes were also upregulated at this time (Figure 6A). OsIDS1 was found to be localized in the cytosol (Figure 6). Other homologous MTs may also be localized in the cytosol like OsIDS1. Induced MT proteins in rice roots would bind the free Zn, Cu, and possibly Mn ions in the cytosol. The induction of *IDS1* and *OsIDS1* by long-term Fe deficiency in barley and rice (Okumura et al. 1991) was thought to reduce stress caused by Zn, Cu, and Mn accumulation during prolonged Fe deficiency. However, heavy metal balance would fluctuate with the onset of Fe deficiency. Given that IDEF1 activity is controlled by the balance of heavy metals (Kobayashi et al. 2011), the strong induction of the MT genes at 6 h of Fe deficiency indicated a change in the balance of heavy metals. Changes in expression of MTs suggested that a fluctuation in heavy-metal availability occurred and was sensed in roots at the very early stage of Fe deficiency. Then, after 24 h of Fe deficiency, Fe uptake and translocation were enhanced by the up regulation of MAs-related genes. The up regulation of *OsYSL13* at 36 h (Figure 5A) suggests its relationship to translocating Fe and/or other heavy metals, since *OsYSL13* was mainly expressed in the root cortex rather than the epidermis or exodermis under Fe-deficient conditions (Inoue et al. 2009), while no substrates of OsYSL13 have been identified. Interestingly, *OsCOPT7* was also upregulated at 36 h with a change in expression similar to MAs-related genes (Table 1). Reports that OsCOPT7 could take up Cu in yeast cells (Yuan et al. 2011) and was localized to tonoplasts in rice roots based on proteomics analysis (Whiteman et al. 2008), suggest that *OsCOPT7* transported Cu in vacuoles to the cytosol. In this respect, the reason for the up regulation of *OsCOPT7* at 36 h remains unclear because Fe uptake was already upregulated and the uptake of Fe and other heavy metals including Cu into root cells may be enhanced. OsCOPT7 might be required for the enhancement of Fe availability or MAs biosynthesis in root cells.

### Early Fe deficiency-inducible genes were regulated in multiple pathways

Early in Fe deficiency, the expression of the upregulated genes showed various patterns. Group A was upregulated only at 3 h (Figure 1) and includes many genes encoding stress-related proteins such as pathogenesis-related proteins and heat shock proteins (data not shown). Up regulation of genes in this group may be a response to the mechanical stress of Fe-deficiency treatment, although some may be directly involved in the Fe-deficiency response. *OsIDS1* was one of the most rapidly induced genes among the investigated Fe deficiency-induced genes (Additional file 3). Since *OsIDS1* is downregulated in *OsIRO2* RNAi plants, *OsIDS1* induction under Fe-deficient conditions is thought to be partially regulated through an OsIRO2 regulation cascade (Ogo et al. 2007). There is no OsIRO2 binding sequence (CACGTGG) in the *OsIDS1* promoter region (Ogo et al. 2007), indicating that the rapid induction of *OsIDS1* at the onset of Fe-deficiency stress may depend on a regulation mechanism different from OsIRO2. The rapid induction of *OsIDS1* at the onset of Fe-deficiency stress might result from a signal occurring at an early step in the Fe-deficiency signal cascades, and/or from the signals of heavy-metal disorder.

The ratio changes and transitions of the signal intensities under each condition were both very alike among the MAs-biosynthetic genes, Met cycle-related genes, *OsYSL15*, *OsIRO2*, and others. This indicates that these genes are regulated by very similar factors in early Fe deficiency as well as under control conditions of Fe sufficiency. Previously, we elucidated the functions of IDEF1, IDEF2, and OsIRO2 in the gene regulation system through their abilities to bind particular sequences and their roles in plant tolerance tests under Fe-deficient conditions (Kobayashi et al. 2007,2009; Ogo et al. 2007,2008,2011). Although IDEF1, IDEF2, and OsIRO2 regulate the expression of MAs-related genes and other Fe deficiency-inducible genes, their binding sequences are not consistently present in upstream regions of the genes that showed similar expression patterns in the current work (Table 2). In addition, we showed that detailed expression patterns of MAs-biosynthetic genes and *OsIRT2* (group B in Figure 1), or for *OsSAMS2* and *OsIRT1* (group C in Figure 1), were not alike (Additional file 3, Figures 3B and 5A). *OsIRT1* and *OsIRT2* were thought to be differently regulated from MAs-biosynthetic genes in early Fe deficiency. Accordingly, our gene profiling in early Fe deficiency strongly suggests that the induction of genes by Fe deficiency is not begun by a single pathway in the regulation network, but involves multiple pathways and multiple components of activators even in early Fe deficiency. The differences in pathways and activator components that trigger induction by Fe deficiency probably influences the timing of the induction of the different genes, such as *OsIRT1* and *OsIRO2*, which are under the regulation of IDEF1 (Kobayashi et al. 2007). In particular, the synchronously expressed group that includes MAs-biosynthetic genes may have the same combination of (known and unknown) *cis*-acting elements that are bound by the same types of transcription factors. The present study of the gene profile of early Fe deficiency provides important information to further elucidate the regulation network of Fe deficiency.

**Table 2:** **Promoter analysis of genes with similar expression patterns during the early stages of Fe deficiency**

	Gene locus	Definition	Number of the binding site		
			IDEF1	IDEF2	OsIRO2
**MAs-related genes**					
	Os03g0307300	OsNAS1	2	4	0
	Os03g0307200	OsNAS2	5	7	0
	Os02g0306400	OsNAAT1	1	5	0
	Os03g0237100	OsDMAS1	0	4	0
	Os01g0323600	OsSAMS2	0	2	0
	Os06g0112200	MTN	1	4	0
	Os04g0669800	OsMTK1	2	1	0
	Os04g0669900	OsMTK2	1	4	0
	Os11g0216900	OsIDI2	0	5	0
	Os11g0484000	DEP	2	2	0
	Os03g0161800	OsIDI1/OsARD2	5	4	0
	Os09g0453800	OsIDI4	3	5	0
	Os12g0589100	OsAPT1	1	6	2
	Os02g0714600	PRPPS	2	0	0
	Os04g0306400	RPI	2	4	0
	Os06g0486800	FDH	2	3	0
	Os11g0134900	TOM1	9	7	0
	Os02g0650300	OsYSL15	1	4	2
**Genes in Table** 1					
	Os06g0639800	Cytochrome P450 family protein	2	5	0
	Os08g0562100	Sorghum chloroplast malate dehydrogenase-like (Fragment)	3	5	0
	Os09g0536700	Nodulin-like domain containing protein	0	3	0
	Os10g0440000	Cytochrome P450 family protein	2	1	0
	Os01g0878700	Amino acid transporter family protein	3	3	0
	Os03g0828600	Sodium/hydrogen exchanger family protein	5	6	0
	Os07g0258400	OsNRAMP1	2	7	0
	Os09g0440700	Leucine-rich repeat protein	1	2	0
	Os09g0345300	OsCOPT7	1	2	0
	Os12g0132500	TOM1-like	10	7	0
	Os01g0952800	OsIRO2	5	0	0
	Os01g0546100	DUF6 containing protein	1	3	1
	Os01g0647200	Non-protein coding transcript	0	6	1
	Os02g0445100	Auxin-responsive family protein-like	2	5	0
	Os03g0204900	Hypothetical protein	1	3	0
	Os03g0256200	Hypothetical protein	10	8	0
	Os07g0253600	(No Hit)	3	3	0
	Os08g0291000	(No Hit)	0	0	0
	Os04g0675000	DUF789 containing protein	2	4	0
	Os05g0519300	DUF506 containing protein	1	6	3
	Os09g0130300	Conserved hypothetical protein	0	8	0
	Os11g0129600	ELM domain containing protein	3	3	0
	Os12g0126200	ELM domain containing protein	1	1	0
	Os12g0236100	Conserved hypothetical protein	1	1	0

Both strands of 1000-nucleotide upstream sequences were examined for the presence of the core binding sequences of three transcription factors. The sequences searched for were CATGC for IDEF1, CA (A/C)G(T/C)(T/C/A)(T/C/A) for IDEF2, and CACGTGG for OsIRO2.

## Conclusions

We showed that many genes were induced in the early stages of Fe deficiency. Among these upregulated genes, MAs-related genes and several other genes such as *OsIRO2* and *OsNRAMP1* were found to be expressed with very similar patterns of regulation. These genes may be regulated by the same combination of known and unknown *cis*-acting elements and the same types of transcription factors.

## Methods

### Plant material

Rice (*Oryza sativa* L. cv. Nipponbare) plants were grown hydroponically in a growth chamber (day 30°C, 14 h; night 25°C, 10 h). The 1× concentration of the nutrient solution for hydroponic culture consisted of 2 mM Ca(NO_3_)_2_, 0.7 mM K_2_SO_4_, 0.1 mM KCl, 0.1 mM KH_2_PO_4_, 0.5 mM MgSO_4_, 0.1 mM Fe(III)–EDTA, 10 μM H_3_BO_3_, 0.5 μM MnSO_4_, 0.5 μM ZnSO_4_, 0.2 μM CuSO_4_, and 0.01 μM (NH_4_)_6_Mo_7_O_24_. Seeds were sown on paper towels wet with ion-exchanged water. Then, the germinated seeds were grown on a net floating on 1× nutrient solution prior to transplanting to hydroponic boxes. The concentration of the nutrient solution was increased later to 1.25× according to the plant growth. The nutrient solution was changed once per week and its pH adjusted to 5.3–5.5 daily. Fe-deficiency treatment was performed at 1 month after germination. On the day before the Fe-deficiency treatment, the solutions of all plants were changed, and the Fe-lacking solution was also prepared at the same time so that the water temperature could equalize. Half of the plants were transferred to the Fe-lacking nutrient solution as the Fe-deficiency group after washing the roots with ion-exchanged water at the beginning of the day. The rest of the plants were grown in the Fe-sufficient nutrient solution as the control group. The pH of the solution for the control group was adjusted at 0 h and after the 24-h sampling. Nine plants from both groups were sampled at 3, 6, 9, 12, 24, and 36 h after the onset of the treatment (Additional file 1).

### Oligo microarray experiment

The rice 44 K oligo DNA microarray (Agilent Technologies, Santa Clara, CA, USA) contained 43,733 DNA probes, which covers about 24,000 assumed genes and micro RNAs. Total RNA was extracted from roots of each sample using Plant RNeasy Kit (Qiagen, Valencia, CA, USA). The microarray experiment, including the labeling of samples, hybridization, and detection, was performed as previously reported (Ogo et al. 2007). For each combination of the control and Fe-deficient groups, a color swap of Cy5 and Cy3 was performed. The two values of signal intensities resulting from the color swap were averaged, and the signal intensities were used to calculate the expression ratio (the averaged signal intensity of the Fe-deficient group divided by that of the control group).

### Analysis of genes up- and down regulated by Fe deficiency

To search for upregulated genes, first, the genes whose expression ratios were over 1.95 at any of the six time points were selected. The genes with signal intensities (under 180 in the induced state) that were too low were excluded, except for genes that showed extremely high expression ratios (over 18). By these criteria, 1003 gene loci represented by 1396 probes were defined as upregulated genes. To search for downregulated genes, genes whose expression ratios were under 0.54 at any of the six time points were selected after excluding the upregulated genes. The genes with signal intensities (under 200 in the control group) that were too low were excluded from further analysis. In total, 325 gene loci represented by 432 probes were defined as down regulated genes.

Many gene loci had multiple corresponding DNA probes on the array. Of the upregulated genes represented by multiple probes on the array, 54 showed varying expression patterns among the probes (Figure 1). The rest of the genes represented by multiple probes showed consistent expression patterns among the probes. For example, three probes on the array corresponded to the Os11g0216900 gene (*IDI2*). Two of the probes showed an expression pattern belonging to group B, and one probe showed expression belonging to group C (see Additional file 4). In such cases, different patterns were counted as representing different genes. Each result shown in the figures and tables was derived from data from one probe. In the case of genes with multiple probes, the result for the probe with the greater signal intensity was chosen. All numerical data, including those from other probes of the same gene locus, are presented in Additional file 4: Table S1. Some probes that satisfied our criteria for selection at one time point had an expression ratio ≥1.95 with low signal intensity (under 180 in the induced state) at another time point (*e.g.*, Os06g0639800 in Table 1). In classifying the upregulated genes (Figure 1), such data for certain time points for some genes were also classified as upregulated time points. In other results, the unsatisfied data are shown with notes.

### Vector construction and transient expression of GFP-fusion proteins

The open reading frame (ORF) of *OsIDS1* was amplified by PCR from a cDNA pool made from Fe-deficient rice roots. The primers used were 5′- tgtacaggaggagtcgacATGTCTTGCTGCGGAGG-3′ and 5′-gtcgactcctcctccGCAGTTGCAGGGATTGC-3′. The amplified fragments were introduced into pCR4-Blunt (Invitrogen, Carlsbad, CA, USA) and the sequence was verified. Then, they were subcloned into the *Sal* I site in a modified plasmid designated CaMV35S-*Sal* I-*Kpn* I-*sGFP* (S65T)-NOS3′ (Ishimaru et al. 2006), in which the target proteins are fused to the N-terminus of sGFP. The orientation of the subcloned fragment was checked by digestion with two restriction enzymes, *Sal* I and *Xho* I, sites that are present in the ORF of *OsIDS1*. These steps resulted in the construction of p35S-OsIDS1-sGFP.

The vectors p35S-sGFP (kindly provided by Dr. Y. Niwa at the University of Shizuoka, Shizuoka, Japan) and p35S-OsIDS1-sGFP were introduced into onion epidermis cells and stomatal cells of *Commelina communis* L. using the Biolistic PDS-1000/He Particle Delivery System (Bio-Rad, Hercules, CA, USA) following the manufacturer’s instructions. After 4 h of incubation at room temperature, the fluorescence of the sGFP fusion proteins was imaged using a confocal microscope (LSM5 Pascal; Carl Zeiss, Tokyo, Japan) equipped with an argon laser and a GFP filter set.

### Promoter analysis

IDEF1, IDEF2, and OsIRO2 binding sequences were searched for within the 1000 nucleotides upstream of the predicted transcriptional initiation sites of the genes. The rice genome sequences were obtained from the Rice Annotation Project Database. The sequences searched for were CATGC for IDEF1, CA (A/C)G(T/C)(T/C/A)(T/C/A) for IDEF2, and CACGTGG for OsIRO2.

## Electronic supplementary material

Additional file 1:**Plant growth and sampling conditions.** A) Rice plants were germinated and grown hydroponically in a growth chamber under conditions of 14 h light at 30°C and 10 h darkness at 25°C. Roots of control and Fe-deficient (−Fe) plants were harvested at 3, 6, 9, 12, 24, and 36 h after the onset of Fe-deficiency treatment. B) Sampling schedule. Fe-deficiency treatment was begun coincident with the start of illumination. Black triangles indicate the sampling time. (PPT 124 KB)

Additional file 2: Stable expression of *IDEF1* and *IDEF2* during Fe-deficiency. (PPT 140 KB)

Additional file 3:**Expression patterns of the previously reported Fe deficiency-inducible genes.** The groups and expression patterns are as in Figure 1. The first report(s) on Fe-deficiency inducibility are given as references. (XLSX 16 KB)

Additional file 4:**Microarray data for genes presented in the figures and tables.** Expression ratios in italics did not meet the selective criteria for signal intensities even when they were greater than 1.95. The results for probes with an asterisk (*) were used in the figures and tables. (XLS 71 KB)

Below are the links to the authors’ original submitted files for images.

Authors’ original file for figure 1

Authors’ original file for figure 2

Authors’ original file for figure 3

Authors’ original file for figure 4

Authors’ original file for figure 5

Authors’ original file for figure 6
