# Supplementary material for: Rice genes involved in phytosiderophore biosynthesis are synchronously regulated during the early stages of iron deficiency in roots
Source: Rice (N Y). 2013 Jun 25;6:16. doi: 10.1186/1939-8433-6-16 (PMC4883707; doi:10.1186/1939-8433-6-16)
Supplement: Supplementary file 1 — Additional file 1:Plant growth and sampling conditions. A) Rice plants were germinated and grown hydroponically in a growth chamber under conditions of 14 h light at 30°C and 10 h darkness at 25°C. Roots of control and Fe-deficient (−Fe) plants were harvested at 3, 6, 9, 12, 24, and 36 h after the onset of Fe-deficiency treatment. B) Sampling schedule. Fe-deficiency treatment was begun coincident with the start of illumination. Black triangles indicate the sampling time. (PPT 124 KB) [file 12284_2012_53_MOESM1_ESM.ppt]

## Slide 1
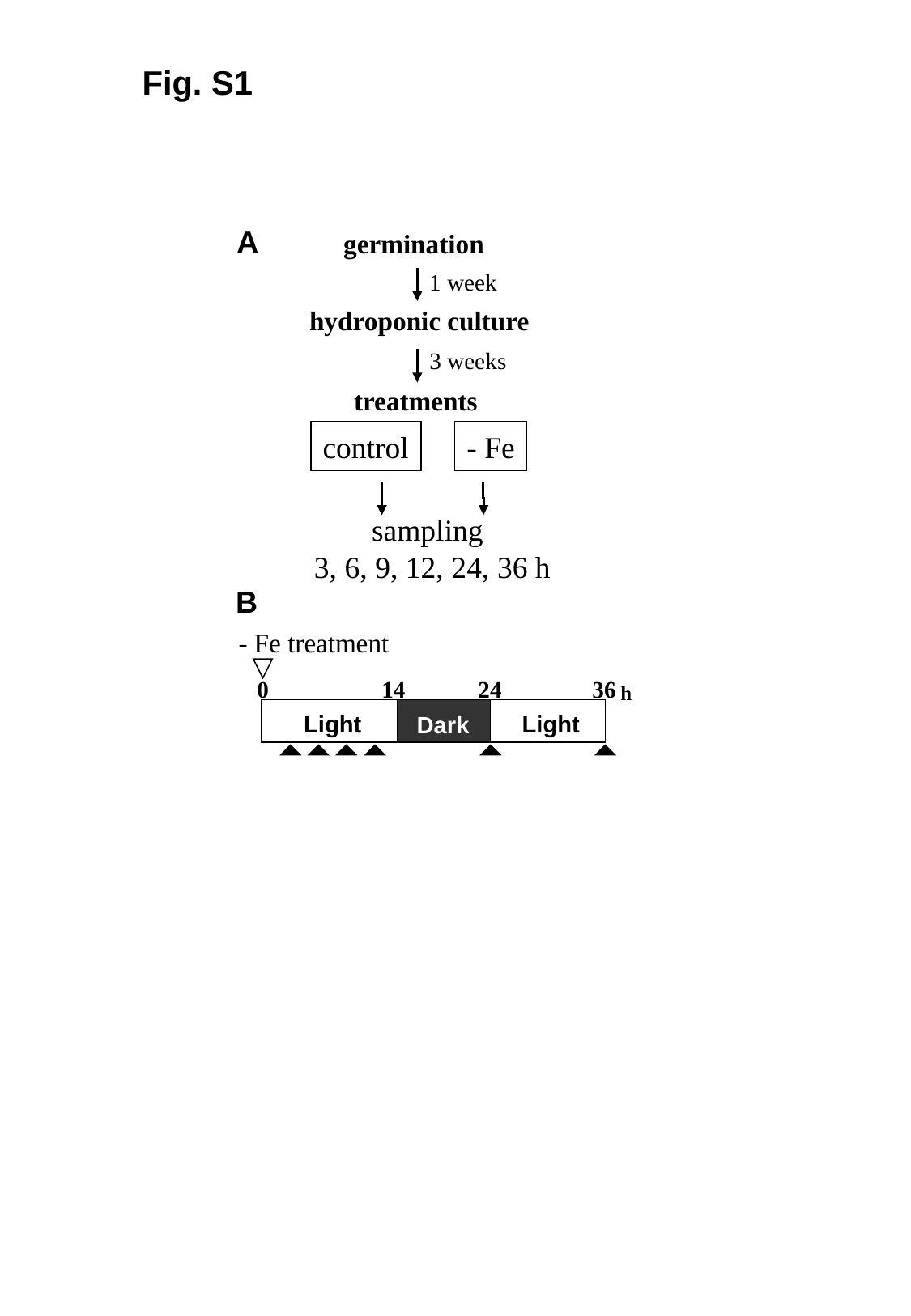

Fig. S1
A
germination
1 week
hydroponic culture
3 weeks
treatments
control
- Fe
sampling
3, 6, 9, 12, 24, 36 h
B
- Fe treatment
0
14
24
36
h
Light
Light
Dark
