# Supplementary material for: Rice genes involved in phytosiderophore biosynthesis are synchronously regulated during the early stages of iron deficiency in roots
Source: Rice (N Y). 2013 Jun 25;6:16. doi: 10.1186/1939-8433-6-16 (PMC4883707; doi:10.1186/1939-8433-6-16)
Supplement: Supplementary file 2 — Additional file 2: Stable expression of IDEF1 and IDEF2 during Fe-deficiency. (PPT 140 KB) [file 12284_2012_53_MOESM2_ESM.ppt]

## Slide 1
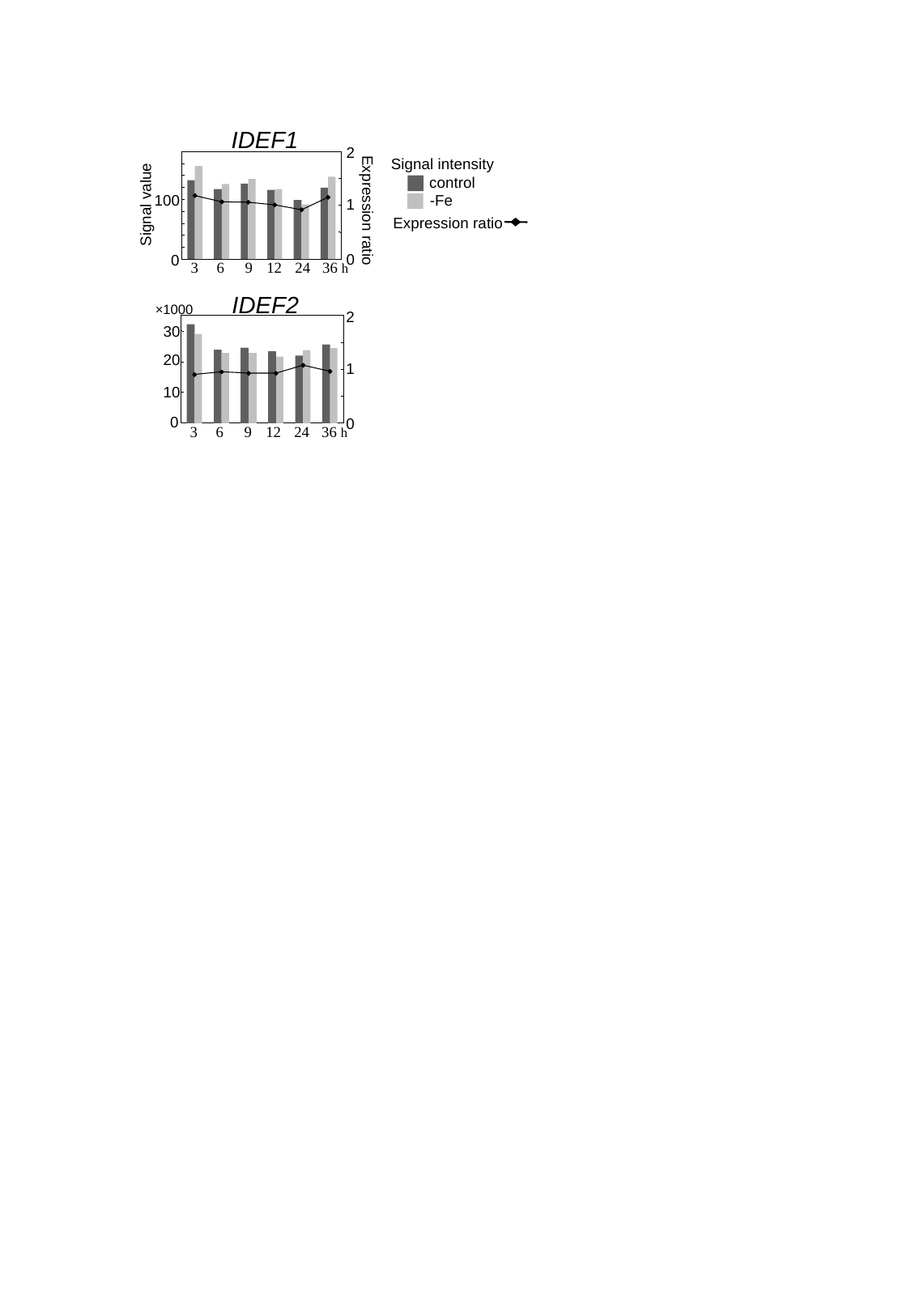

IDEF1
2
Signal intensity
control
100
Signal value
-Fe
1
Expression ratio
Expression ratio
0
0
3
6
9
12
36 h
24
IDEF2
×1000
2
30
20
1
10
0
0
3
6
9
12
36 h
24
