# Supplementary material for: Rice genes involved in phytosiderophore biosynthesis are synchronously regulated during the early stages of iron deficiency in roots
Source: Rice (N Y). 2013 Jun 25;6:16. doi: 10.1186/1939-8433-6-16 (PMC4883707; doi:10.1186/1939-8433-6-16)
Supplement: Supplementary file 5 — Authors’ original file for figure 1 [file 12284_2012_53_MOESM5_ESM.pdf]

Expression pattern	3	6	9	12	24	36 (h)	gene number
A	■						257
B					■	■	96
C						■	85
D					■		63
E	■	■	■	■	■	■	51
F	■	■					39
G	■	■	■	■	■		36
H				■			32
I		■					31
J	■	■	■				25
Other 51 patterns							353
Total							1068
