# Supplementary figures and images for: Rice genes involved in phytosiderophore biosynthesis are synchronously regulated during the early stages of iron deficiency in roots
Source: Rice (N Y). 2013 Jun 25;6:16. doi: 10.1186/1939-8433-6-16 (PMC4883707; doi:10.1186/1939-8433-6-16)

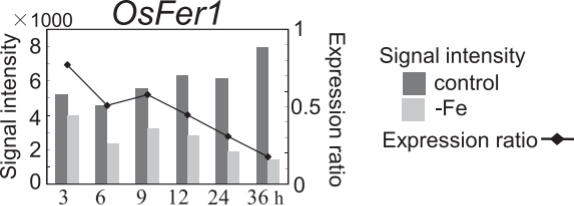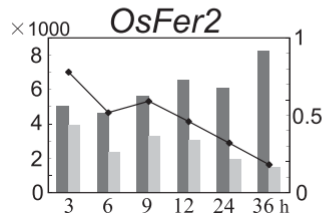

Supplement: Supplementary file 6 — Authors’ original file for figure 2 [file 12284_2012_53_MOESM6_ESM.pdf]

**A**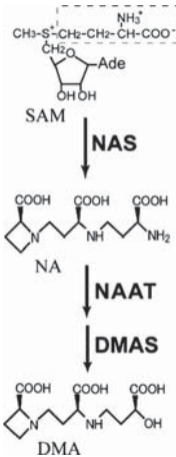**B**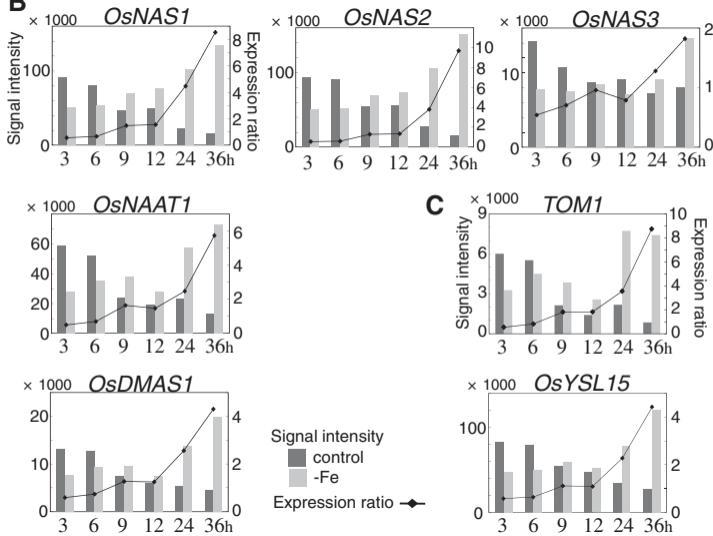**C**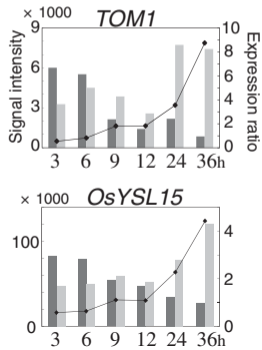

Supplement: Supplementary file 7 — Authors’ original file for figure 3 [file 12284_2012_53_MOESM7_ESM.pdf]

**A**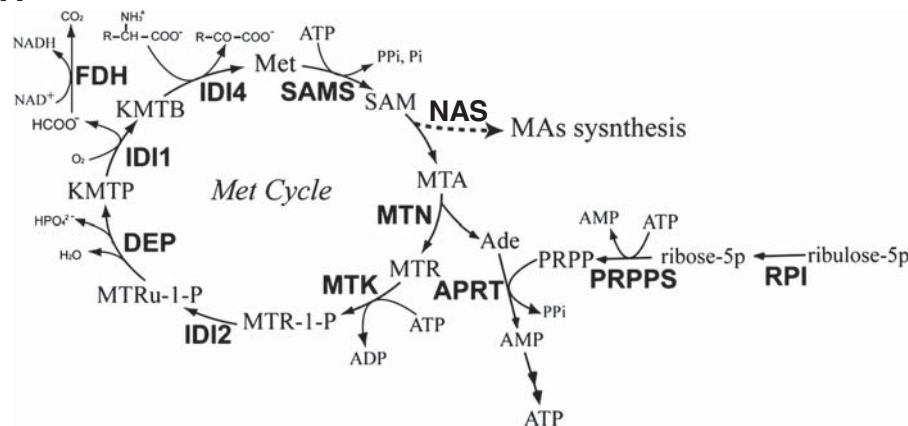**B**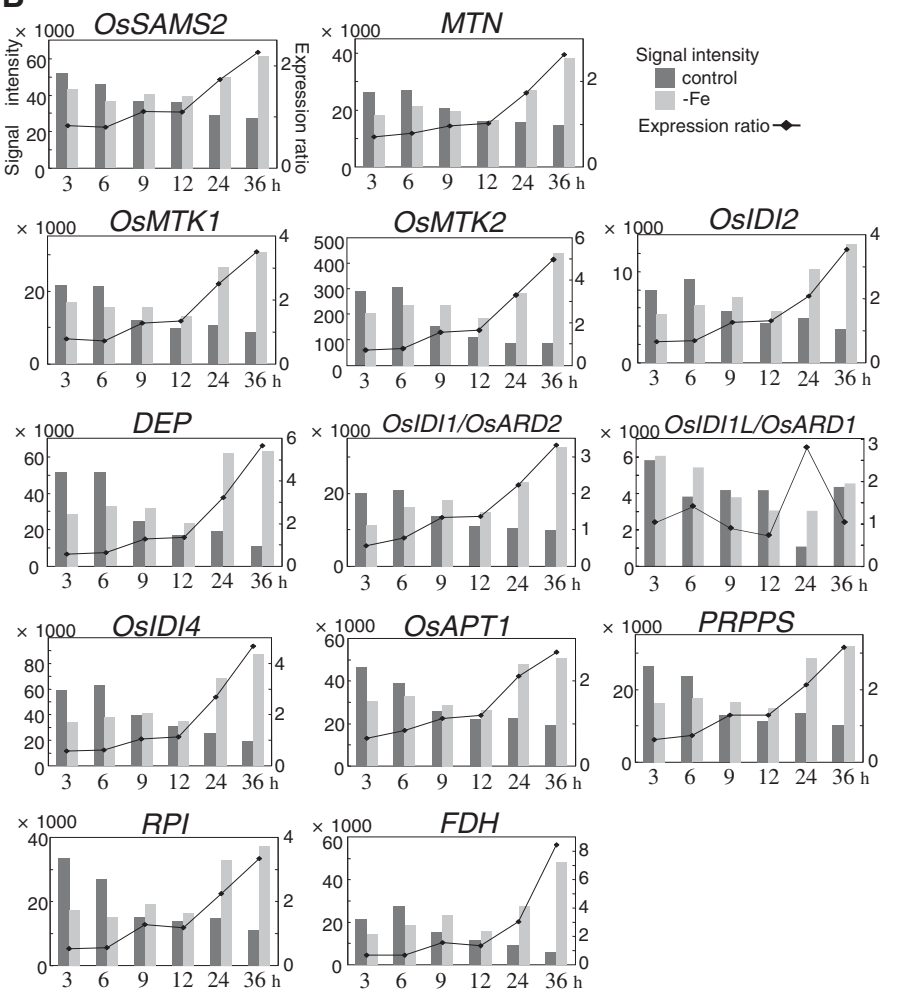

Supplement: Supplementary file 8 — Authors’ original file for figure 4 [file 12284_2012_53_MOESM8_ESM.pdf]

**A**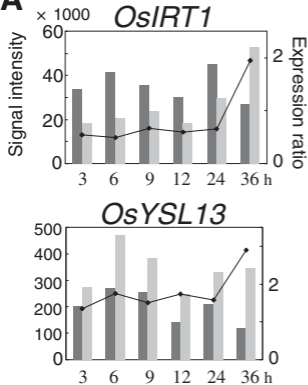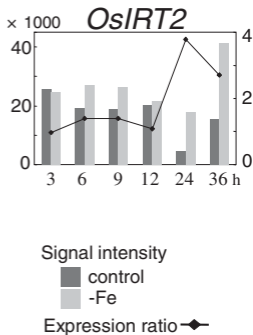**B**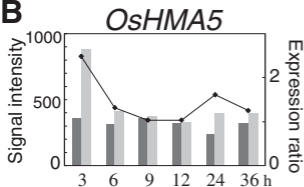**C**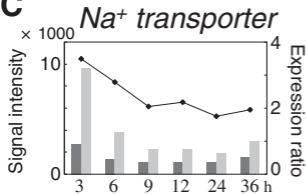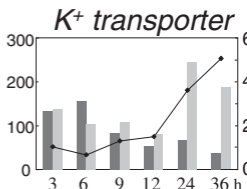

Supplement: Supplementary file 9 — Authors’ original file for figure 5 [file 12284_2012_53_MOESM9_ESM.pdf]

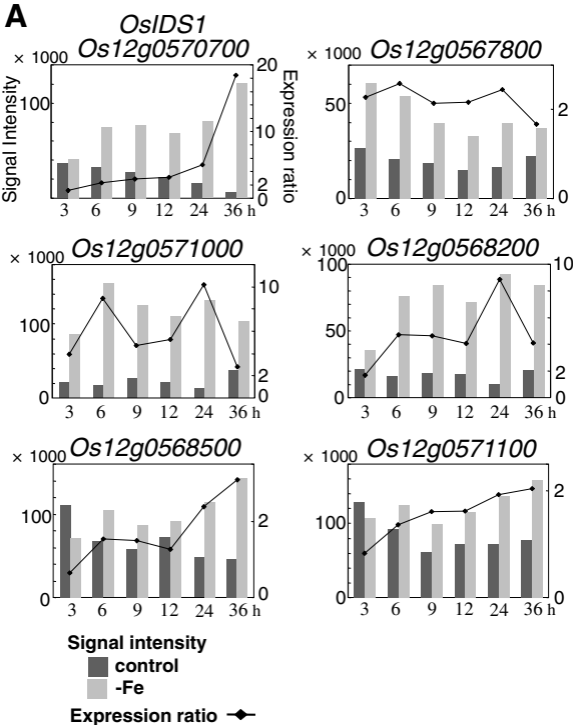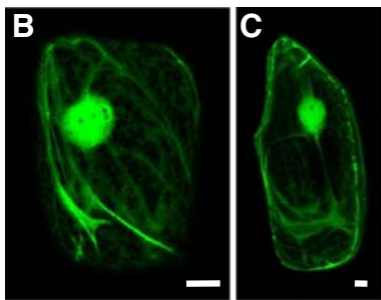

Supplement: Supplementary file 10 — Authors’ original file for figure 6 [file 12284_2012_53_MOESM10_ESM.pdf]
